# Protocatechualdehyde Induced Breast Cancer Stem Cell Death via the Akt/Sox2 Signaling Pathway

**DOI:** 10.3390/ijms26051811

**Published:** 2025-02-20

**Authors:** Seung-Yeon Ko, Seonghee Park, Youn-Hee Choi

**Affiliations:** 1 Department of Physiology, College of Medicine, Ewha Womans University, Seoul 07804, Republic of Korea; kosy8626@ewha.ac.kr (S.-Y.K.); sp@ewha.ac.kr (S.P.); 2 Inflammation-Cancer Microenvironment Research Center, College of Medicine, Ewha Womans University, Seoul 07804, Republic of Korea

**Keywords:** cancer stem cell, breast cancer, protocatechualdehyde, Akt, Sox2, ALDH1A

## Abstract

Breast cancer (BC) is most frequently recognized in women and characterized by histological and molecular heterogeneity. Among the various subtypes, triple-negative BC remains the most challenging disease owing to the lack of effective molecular targets and the high frequency of breast cancer stem cells (BCSCs), which account for both recurrence and resistance to conventional treatments. Despite the availability of hormonal therapies and targeted treatments, patients still face early and late relapses, necessitating new cytotoxic and selective treatment strategies. Our study focuses on investigating the effects of protocatechualdehyde (PCA), a potent bioactive compound derived from *Artemisia princeps*, on CSCs in BC cells. PCA inhibited BC growth and mammosphere formation as the concentration increased. This agent decreased the fraction of the CD44^+^/CD24^−^ population, the aldehyde dehydrogenase 1A-expressing population, and the protein level of Sox2 in breast CSCs by downregulating Akt and pAkt. Moreover, PCA treatment reduced the tumor volume and weight in 4T1-challenged BALB/c mice. Collectively, our findings support the anti-tumor effect of Akt/Sox2-targeting PCA, suggesting a novel utilization of PCA in BC therapy.

## 1. Introduction

Breast cancer (BC) is one of the leading causes of cancer death among women, primarily because of metastasis and recurrence [1,2,3]. BC can be categorized into four subtypes of breast cancer: luminal A, luminal B, HER2-enriched, and triple-negative breast cancer (TNBC) [4]. Among these, TNBC comprises 15–20% of all BC cases and is related to a particularly dismal prognosis [5]. Despite significant advancements in therapeutic interventions, BC recurrence and metastasis continue to contribute to high mortality rates.

A critical factor in BC progression is the presence of cancer stem cells (CSCs), a small distinct subpopulation within tumor tissues with self-renewal ability, drive drug resistance and contribute to relapse and metastasis [6]. Since CSCs derived directly from tumor tissues are difficult to obtain, researchers have turned to CSCs derived from BC cell lines [7]. Notably, cancer cells expressing aldehyde dehydrogenase 1A1 (ALDH1A1), a CSC marker, have been identified as key contributors to poor patient outcomes [8].

Breast CSCs have been successfully isolated both from patient samples following in vitro propagation and from established BC cell lines [9,10]. CSCs can self-renew and differentiate into diverse phenotypes, thus sustaining tumor growth and heterogeneity [11]. Targeting these CSCs represents a promising therapeutic approach, particularly for TNBC [12,13], for which treatment options remain limited.

Cancer stemness is recognized by the presence of stemness-related markers in human cancers such as Oct4, Sox2, Klf4, c-Myc, Sall4, and Nanog [14]. The Akt signaling has been related to control CSC properties, serving as an upstream activator of Oct4 and other stemness markers [15,16]. Akt phosphorylation promotes CSC formation by upregulating Oct4 and Nanog expression [15,17]. Moreover, RNA interference-mediated knockdown of Akt results in decreased Oct4 expression and suppression of CSC characteristics, offering insights into potential therapeutic strategies targeting this pathway [18].

*Artemisia princeps* is a member of the Asteraceae family of plants, which consists of 500 widely distributed species. *Artemisia princeps* is a well-known traditional herbal medicine that is mainly found in Korea, China, and Japan. The plant leaf is often used as a tea and has traditionally been utilized to treat inflammation, diarrhea, gastric ulcers, and circulatory disorders [19]. Leaves of *Artemisia* contain a higher content of flavonoids, caffeoylquinic acids, and monoterpenoids, which possess various bioactivities, including antimalarial, antiviral, antioxidant, and anti-cancer effects [20,21,22]. Recently, anti-inflammatory effects in an atopic dermatitis model were enhanced by fermenting *Artemisia capillaris* leaves using *Ganoderma lucidum* in a solid-state fermentation [23]. An ethyl acetate fraction of *Artemisia capillaris* (AC68) exerts an anti-cancer activity by suppressing the PI3K pathway in hepatocellular carcinoma (HCC) [24]. Protocatechualdehyde (PCA) exerted anti-cancer activity by reducing the protein level of cyclin D1 through the regulation of HDAC2 in colorectal cancer cells. In breast cancer cells, PCA reduced the protein level of cyclin D1 independent of β-catenin degradation [25,26].

This study focused on investigating the anti-CSC properties of *Artemisia* fermentation, and a purified compound isolated through bioactivity-guided fractionation and chromatography in BC cells and CSCs. Specifically, we investigated the anti-CSC activity of protocatechualdehyde (PCA), a purified compound derived from *Artemisia* fermentation, and elucidated the molecular mechanisms underlying its anti-cancer effects.

## 2. Results

### 2.1. Isolation of a CSC Inhibitor Derived from Artemisia Princeps Fermentation Using Lactobacillus rhamnosus

**Figure 1:**
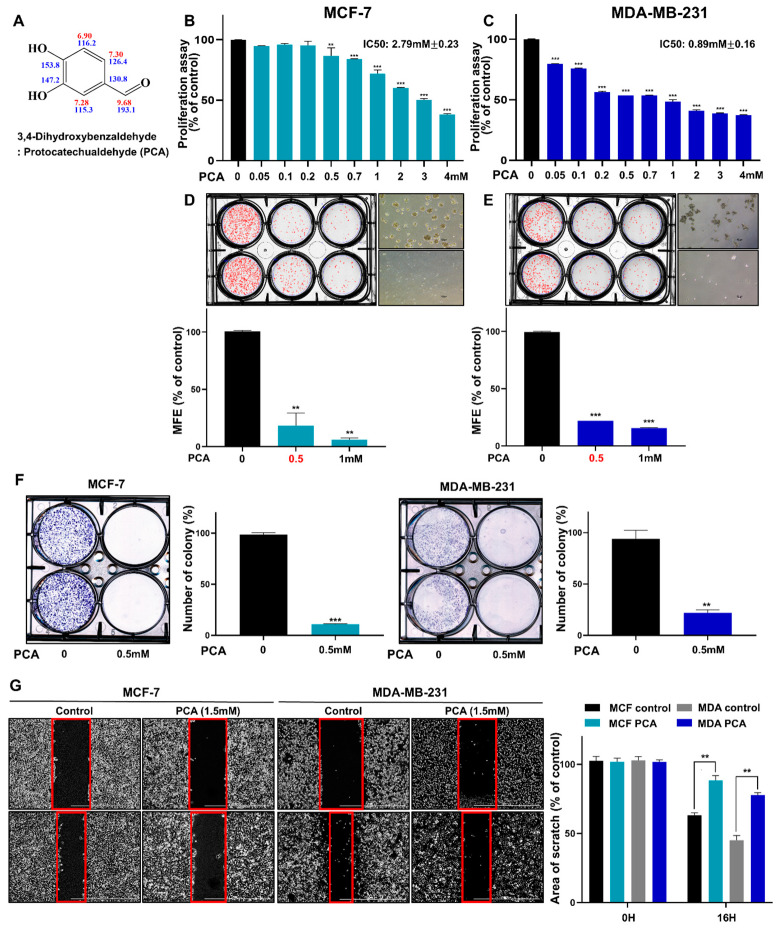
Effect of PCA on the proliferation of MCF-7 and MDA-MB-231 breast cancer cells and mammosphere formation. (**A**) Molecular structure of PCA. (**B**,**C**) Anti-proliferative effects of PCA on MCF-7 and MDA-MB-231 breast cancer cells were assessed using WST assays after treatment with increasing PCA concentrations. (**D**,**E**) Mammosphere formation was evaluated in MCF-7 and MDA-MB-231 cells incubated with PCA in CSC culture media for 7 days (scale bar: 100 μm). (**F**) PCA repressed colony formation in MCF-7 and MDA-MB-231 cells cultured for 7 days. (**G**) The effect of PCA on cell migration was assessed in BC cell lines, with migration captured at 0 and 16 h. The inhibition rate of migration was measured relative to untreated controls. Data are representative of at least three independent experiments. ** *p* < 0.01, *** *p* < 0.001 vs. control.

To isolate the CSC inhibitor derived from *Artemisia princeps* fermentation with *Lactobacillus rhamnosus* and MRS media containing 2% *Artemisia* powder, we analyzed the HPLC data of extracts obtained from the Lactobacillus broth after 0 and 5 days of culture with ethyl acetate extraction (cultured broth:EtOAc = 1:1). Our data showed one different peak in the red box. To identify breast CSC inhibitors, we conducted an assay to determine the ability to form mammospheres using the red-box fraction. This fraction also inhibited the formation of mammospheres (Figure S1). This showed that this fraction included a CSC inhibitor. The isolated material was verified using HPLC and TLC (Figures S2–S6). Figure S7 presents an overview of the bioassay-guided isolation procedure. The fraction was purified using ethyl acetate extraction, ODS column chromatography, silica gel chromatography, preparatory TLC, and HPLC. NMR and mass spectrometry were used to identify the molecular structure of the purified sample. The 1H NMR spectra were measured using CD_3_OD. In the 13C NMR spectrum, eight carbon peaks at δ 193, 153.7, 147, 130, 126, 116, 115, and 49 were observed (Figure S8). COSY, HMBC, HMQC, and EMI-mass determined the structure of the active compound to be 3, 4-dihydroxybenzaldehyde, also known as protocatechualdehyde (PCA) (Figure 1A and Figures S8–S11). Using ESI mass data, the molecular mass of the active peak was identified as 138, with a quasi-molecular ion peak at *m*/*z* 137.0 [M-H]^−^ in the negative mode (Figure S12).

### 2.2. PCA Suppresses the Growth of BC Cells and the Formation of Mammosphere

We assessed the anti-proliferative effect of PCA in breast cancer cells using WST. Cell growth was inhibited as the concentration increased by ≥500 µM PCA (MCF-7) and ≥50 µM PCA (MDA-MB-231) after 1 day and ≥50 µM PCA (MCF-7 and MDA-MB-231) after 3 days culture (Figure 1B,C and Figure S13). These results demonstrated that PCA significantly inhibited the growth of BC cell lines. To investigate whether PCA suppressed the formation of mammospheres, we administered PCA to mammospheres derived from BC cells. As shown in Figure 1D,E, PCA inhibited primary mammosphere formation, which derived from BC cell lines. PCA reduced the count and size of mammospheres (Figure 1D,E). PCA treatment suppressed colony formation and migration (Figure 1F,G). Our results showed that PCA suppressed the proliferation, migration, and formation of mammospheres.

### 2.3. PCA Reduced CD44^high^/CD24^low^-Expressing and ALDH+ Cancer Cells

**Figure 2:**
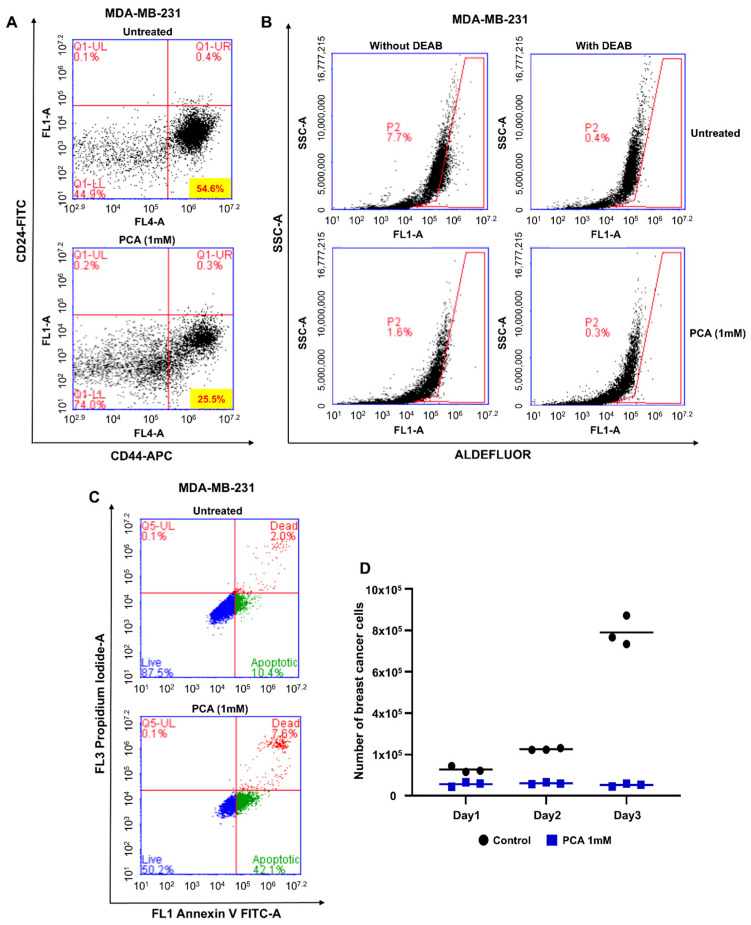
Effect of PCA on the expression of the CSC markers, apoptosis, and mammosphere growth in MDA-MB-231 cells. (**A**) Flow cytometry analysis of CD44^high^/CD24^low^ cells after PCA treatment (1 mM). (**B**) ALDH-positive cell population was assessed using an ALDEFLUOR kit with DEAB as a negative control. The representative flow cytometric scatter plots are shown. The right section displays ALDH-positive cells, while the left section shows ALDH-positive cells without DEAB. The ALDH-positive population is selected in the box. (**C**) PCA-induced apoptosis in mammospheres was assessed via Annexin V/propidium iodide staining. (**D**) Mammosphere growth was significantly reduced by PCA treatment. Single cells from mammospheres were plated and counted over three days.

We examined the effect of PCA on CD44^high^/CD24^low^-expressing and ALDH+-expressing cancer cells. MDA-MB-231 cells were incubated with or without PCA for 24 h, and the populations of CD44^high^/CD24^low^-expressing and ALDH+ cells were determined. The percentage of CD44^high^/CD24^low^-expressing subpopulations decreased from 54.6 to 25.5% after PCA treatment (Figure 2A). PCA-treated cells showed a decrease in ALDH expression from 7.7 to 1.6% (Figure 2B). Therefore, PCA decreased the levels of breast CSC markers.

### 2.4. PCA Treatment Induces BCSC Apoptosis and Suppresses Mammosphere Proliferation

BCSCs were treated with 1 mM PCA to analyze the effect of the compound on apoptosis in mammospheres. The proportion of early apoptotic cells rose from 10.4 to 42.1% (Figure 2C). In addition, PCA decreased BCSC proliferation (Figure 2D).

### 2.5. Effect of PCA on Akt and pAkt Protein Levels in Breast CSCs

**Figure 3:**
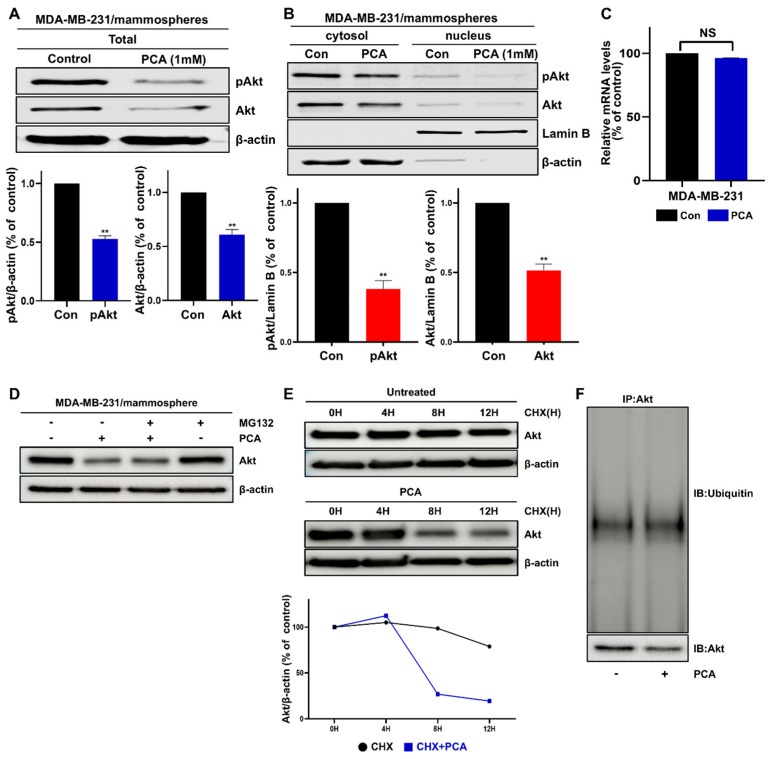
Effect of PCA on the Akt signaling pathway and ubiquitin-independent proteasomal degradation in breast CSCs. (**A**) Immunoblot analysis was examined to quantify Akt and pAkt in cells treated with PCA (1 mM) for 1 day, with β-actin as an internal control. (**B**) The cytosolic and nuclear protein expression and phosphorylation of Akt in mammospheres were analyzed using antibodies for pAkt, Akt, Lamin B, and β-actin. PCA reduced the protein expression of nuclear pAkt and Akt in mammospheres. (**C**) Akt expression was evaluated using real-time RT-qPCR with β-actin as a loading control. (**D**) Immunoblot analysis in mammospheres treated with MG-132 and PCA (1 mM). (**E**) Cycloheximide chase assays measured AKT protein half-life. CHX at 100 µg/mL was used to treat the mammospheres for the designated times, and the stability of endogenous AKT protein was assessed. (**F**) Immunoprecipitation of AKT followed by ubiquitin immunoblotting. Data are representative of at least three independent experiments. ** *p* < 0.01 vs. control.

To determine the targets of PCA, we analyzed the protein expression of Akt and pAkt. PCA reduced the level of total, cytosolic, and nuclear fractions of Akt and pAkt in breast CSCs (Figure 3A,B), but did not decrease Akt transcript levels in breast CSCs (Figure 3C).

### 2.6. PCA Mediates Akt Degradation in a Ubiquitin-Independent Manner

Ubiquitin-proteasomal degradation mainly regulates Akt via E3 ligase [27,28] and ubiquitin-independent proteasomal degradation of Akt occurs [29]. The suppression of Akt signaling reduces the expression of downstream signaling mediators, Oct4, Sox2, and Nanog [17,30,31]. We examined whether Akt downregulation by PCA occurred through ubiquitin-dependent or -independent pathways. Treatment with the proteasome inhibitor MG132 did not protect Akt from PCA-induced degradation (Figure 3D). As shown in Figure 3F, there was no accumulation of ubiquitinated Akt in PCA-treated cells compared to control cells, and the cycloheximide experiment showed that PCA induced the degradation of the Akt protein (Figure 3E,F). Mammospheres were incubated with MG132 and PCA (1 mM) for 1 day and lysed for Western blot analysis for Sox2 protein. The treatment of cells with the proteasome inhibitor MG132 did not protect Sox2 from PCA-induced Sox2 degradation, suggesting that PCA did not enhance the proteasomal degradation of Sox2 (Figure S14). Our results suggested that PCA induces Akt and Sox2 degradation via a ubiquitin-independent pathway.

### 2.7. An Akt-Dependent Mechanism Mediates PCA Suppression of Sox2

**Figure 4:**
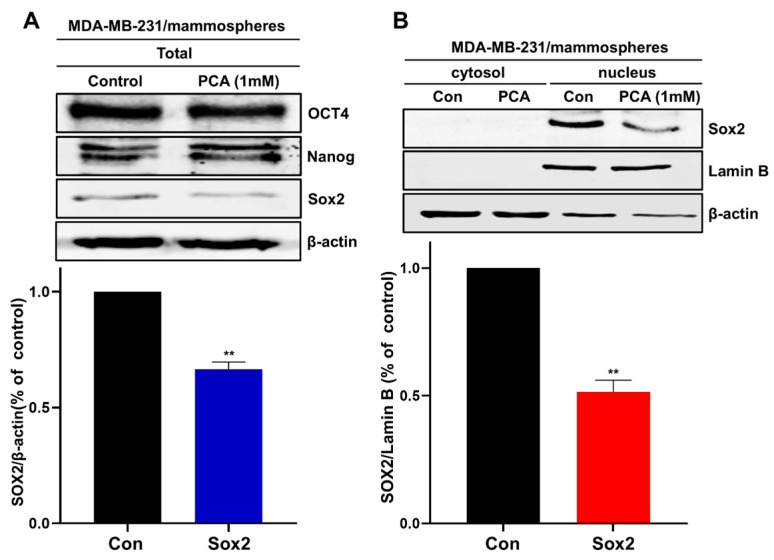
PCA regulates Sox2 in breast CSCs. (**A**) Immunoblot analysis of Sox2, Oct4, and Nanog in mammospheres treated with PCA (1 mM) for 24 h. (**B**) Subcellular distribution of Sox2 in mammospheres analyzed via Western blotting. PCA reduced nuclear and cytosolic Sox2 protein levels. Data are representative of at least three independent experiments. ** *p* < 0.01 vs. control.

CSC stemness involves elevated levels of stemness markers such as Oct4, Sox2, c-Myc, and Nanog [32]. To examine whether CSC inhibition by PCA involved CSC-related transcription factors, mammospheres were treated with 1 mM PCA, and the protein levels of Oct4, Nanog, and Sox2 were determined. As indicated in Figure 4A, Sox2 protein levels were reduced in PCA-treated mammospheres. Because Sox2 is a nuclear protein, we checked the level of nuclear Sox2 protein after PCA treatment. PCA reduced nuclear Sox2 levels compared to the vehicle (Figure 4B). Figure 4A,B show that CSC inhibition may be a consequence of a PCA-dependent reduction in Sox2 expression.

### 2.8. Functional Assessment of PCA as an Anti-CSC Agent in Human HCC1937 Breast Cancer Cells

**Figure 5:**
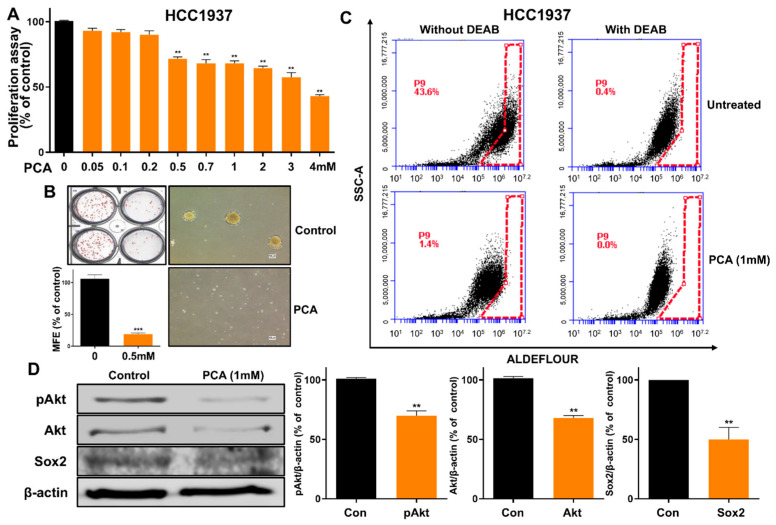
PCA inhibits mammosphere formation in HCC1937 cells. (**A**) WST assay measuring cell viability in HCC1937 cells treated with increasing PCA concentrations for 24 h. (**B**) Mammosphere formation inhibition by PCA (0.5 mM) over 7 days, imaged at 10× magnification. (**C**) ALDH expression levels were evaluated using an ALDEFLUOR assay and flow cytometry after PCA treatment (1 mM). (**D**) Protein expression of pAkt, Akt, and Sox2 in mammospheres treated with PCA (1 mM) for 24 h. Data are representative of at least three independent experiments. ** *p* < 0.01 vs. control.

To confirm the anti-CSC effect of PCA observed in the breast adenocarcinoma cell lines MCF-7 and MDA-MB-231 across different breast cancer cell lines, we next evaluated its efficacy in the ductal carcinoma cell line HCC1937. We examined the effect of PCA as an inhibitor of the Akt/Sox2 signaling in breast CSCs from TNBC and HCC1937 cancer cells. PCA inhibited cell proliferation and mammosphere formation at 0.5 mM (Figure 5A,B). Because ALDH1 is a marker of breast CSCs, we determined the level of ALDH1A in HCC1937 cells treated with PCA. PCA reduced the ALDH1 levels of the HCC1937 subpopulation (Figure 5C). To determine the biochemical effects of PCA on HCC1937, we examined the Akt, pAkt, and Sox2 levels. Our result showed that PCA reduced the total protein levels of Akt, pAkt, and Sox2 (Figure 5D). Thus, PCA reduced breast CSC formation via the Akt/Sox2 signal pathway.

### 2.9. Effect of PCA on Anti-CSCs Using 4T1 Murine BC Cells

**Figure 6:**
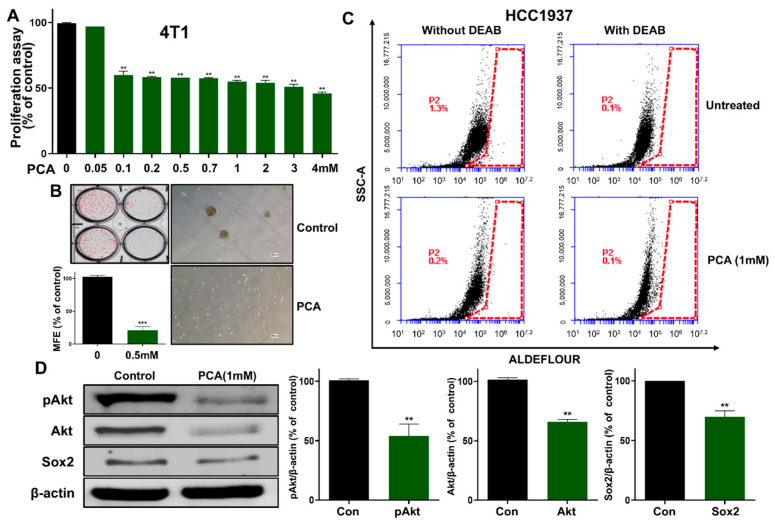
PCA inhibits mammosphere formation in 4T1 cells. (**A**) WST assay of 4T1 cells treated with increasing PCA concentrations for 24 h. (**B**) Mammosphere formation inhibition by PCA (0.5 mM) over 7 days, imaged at 10× magnification. (**C**) ALDH expression in 4T1 cells was assessed using an ALDEFLUOR assay and flow cytometry following PCA treatment (1 mM). (**D**) The expression levels of pAkt, Akt, and Sox2 in mammospheres treated with PCA (1 mM) for 24 h. Data are representative of at least three independent experiments. ** *p* < 0.01, *** *p* < 0.001 vs. control.

Before conducting mouse animal experiments, we performed studies using the murine breast cancer cell line 4T1 to determine whether the anti-CSC effect of PCA observed in human breast cancer cell lines is also effective in murine cells. We examined the effect of PCA as an Akt/Sox2 signaling inhibitor in targeting breast CSCs in 4T1 cancer cells. PCA inhibited cell proliferation and mammosphere formation at 0.5 mM (Figure 6A,B). Because ALDH1 is a breast CSC marker, we examined the levels of ALDH1 protein in 4T1 cells treated with PCA. This compound decreased the ALDH1 levels in the 4T1 subpopulation (Figure 6C). To examine the biochemical effects of PCA on 4T1 cells, we analyzed the Akt, pAkt, and Sox2 levels. Our data indicated that PCA decreased the total protein levels of Akt, pAkt, and Sox2 (Figure 6D). PCA reduced mouse breast CSC formation via the Akt/Sox2 signaling pathway.

### 2.10. Anti-Tumor Effect of PCA in the 4T1 Mouse Model

**Figure 7:**
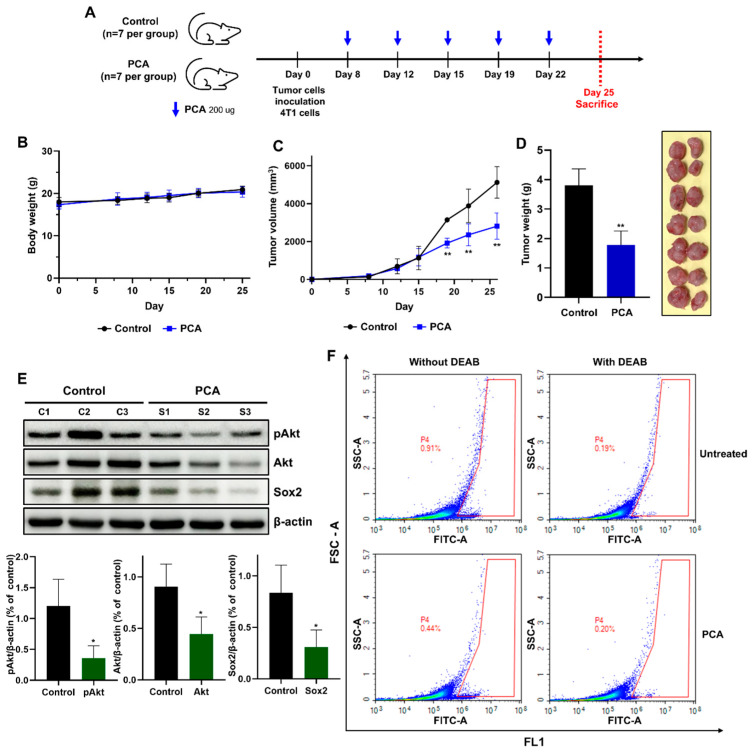
PCA suppresses tumor growth in mouse models. (**A**) The schedule for the in vivo experiment is as follows. (**B**,**C**) Tumor volume and body weight changes in mice injected with 4T1 cells (1 × 10^6^ cells/mouse) and treated with PCA for 25 days. (**D**) Tumor weight was examined after the mice were sacrificed (*n* = 7 per group). (**E**) Western blot analysis of pAkt, Akt, and Sox2 protein levels in tumor samples. (**F**) ALDH expression in tumor-derived single cells was examined using an ALDEFLUOR kit and flow cytometry. Data are representative of at least three independent experiments. * *p* < 0.01, ** *p* < 0.001 vs. control.

The 4T1 mouse cancer cell line derived from BALB/c mouse BC cells can be used as an orthotopic syngeneic breast tumor mouse model to demonstrate the expression of pAkt, Akt, and Sox2. As PCA has anti-proliferative activity in 4T1 cells, we employed a mouse tumor system to investigate whether it represses tumor growth (Figure 7A). There was no change in the body weight of the control and PCA-treated BALB/c mouse model. (Figure 7B). The weights and volumes of tumors were reduced in PCA-treated BALB/c mice compared to the BALB/c control mice (Figure 7C,D). To assess PCA’s effect on 4T1 tumors in vivo, we analyzed the protein expression of the tumor tissues through immunoblot. Compared to the untreated group, there were reduced expressions of the pAkt, Akt, and Sox2 proteins in PCA-treated 4T1 tumor tissues (Figure 7E). Our results showed that PCA significantly inhibited tumor growth. We separated tumors derived from cells and measured the level of the ALDH1 activity using the ALDEFLOUR^TM^ kit. PCA decreased the ALDH1-positive population from 0.91 to 0.44% in 4T1 breast tumors (Figure 7F). Our results indicated that PCA decreased the proportion of ALDH1-expressing subpopulations in tumors, along with breast CSC traits.

## 3. Discussion

PCA, an active compound isolated from *Artemisia* fermentation, effectively inhibited mammosphere formation, demonstrating its anti-CSC activity. Our results showed that PCA performed anti-CSC effects via downregulating the Akt/Sox2 signaling pathway, a critical pathway for CSC survival in BC.

Natural foods have long been known for their potential in cancer prevention and treatment, with certain components in fermented foods and herbal decoctions shown to reduce cancer risk [33,34]. Lactic acid bacteria (LAB) are essential in fermentation by producing various bioactive compounds, including bacteriocins, aroma compounds, exopolysaccharides, bioactive peptides, vitamins, and enzymes [35]. Recent studies have emphasized the antioxidant properties of blackberries, and the anti-CSC activity of catechol derived from aronia juice, both of which are enhanced by LAB fermentation [36,37]. These findings highlight the critical role of CSCs in cancer therapy. In our study, we used *Artemisia* fermentation by *Lactobacillus* to specifically target breast CSCs. *Lactobacillus* strains possess tannase activity, which degrades tannic acid into gallic and protocatechuic acids via ester hydrolysis [38]. Additionally, *Artemisia* species are recognized for their potent bioactive compounds, which have demonstrated therapeutic potential, particularly in hepatocellular carcinoma [22,24]. Based on this, we selected *Artemisia* for screening as a candidate with both anti-cancer and anti-CSC properties in BC. Notably, we are the first to purify PCA, an active compound from *Artemisia* fermentation, and identify its anti-CSC activity.

A breast CSC inhibitor was isolated through LAB fermentation using 2% *Artemisia* powder. PCA, protocatechuic aldehyde, is a natural phenolic aldehyde found in herbs like green bananas, grapevine leaves, barley, and roots of *Salvia miltiorrhiza* [39,40]. PCA exhibits an anti-proliferative effect against colorectal cancer cells by repressing the protein expression of β-catenin, HDAC2, and cyclin D1 [25,26]. Recent research has demonstrated that PCA exerts anti-cancer effects in BC cells via targeting CtBP1 [41]. PCA inhibits cell proliferation and migration via binding to CtBP1, indicating its potential as a specific CtBP1 inhibitor [41]. The druggability of PCA being able to modulate a target protein showed three targets such as C-terminal Binding Protein 1 (CtBP1), nuclear pyruvate kinase M2, and tyrosinase proteins [41,42,43].

Our data provide the first evidence that PCA has not only anti-cancer but also anti-CSC activities for BC treatment. PCA inhibited mammosphere formation in MCF-7 and MDA-MB-231 cells, reduced the size and frequency of mammosphere formation, and induced mammosphere apoptosis. PCA decreased the CD44^high^/CD24^low^ and ALDH1+ subpopulations of breast cancer cells and decreased the protein levels of Akt, pAkt, and Sox2. In the HCC1937 cell line, PCA also suppressed the formation of mammospheres, decreased the ALDH+ subpopulation, and reduced the protein expression levels of pAkt, Akt, and Sox2. Thus, PCA may serve as an anti-CSC agent against BC by reducing stemness.

Several strategies for targeting CSCs have been studied extensively. Natural compounds derived from plant sources have attracted interest in cancer therapy owing to their various therapeutic properties [44]. Vanillin, an active compound found in *Vanilla planifolia,* can be used as a flavor in food and cosmetics [45]. Vanillin reduces the stemness of lung cancer cells by suppressing the Akt/Oct4 signaling pathway [16]. An active compound, catechol, derived from chokeberries and aronia through LAB fermentation inhibits breast CSC formation via the Stat3/IL-6 signaling [37]. This study provides evidence of an active compound, PCA, derived from *Artemisia princeps* through LAB fermentation, significantly inhibiting breast CSC formation through the Akt/Sox2 pathway.

**Figure 8:**
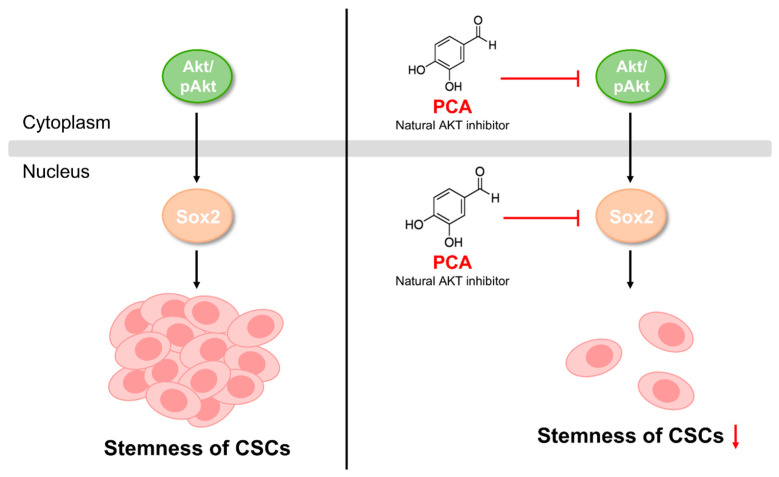
Proposed model of PCA-induced CSC death. The red arrow indicates inhibition of stemness.

In our study, PCA effectively inhibited mammosphere formation by suppressing the Akt signaling pathway. Previous studies have suggested that Akt is crucial for cancer cells and CSC survival [46,47]. Akt blockade inhibits spheroid formation and enhances Oct4 degradation [48]. Diminished Akt function reduces CSC behavior and stem cell marker expression of Oct4 and Nanog [16,49]. Akt is primarily controlled by ubiquitin-dependent proteasomal degradation via E3 ligases [27,28]. We investigated whether PCA-induced downregulation of Akt occurs via a ubiquitin-independent proteasomal degradation pathway. Our results showed that PCA induced Akt degradation, but not via ubiquitin-dependent proteasomal degradation. Proteasomal degradation consists of ubiquitin-dependent and ubiquitin-independent paths. The representative proteins degraded by ubiquitin-independent proteasomes are Rpn4, thymidylate synthase, and ornithine decarboxylase. The ubiquitin-independent degradation pathway is a historic remnant, and ubiquitin-independent degradation may offer an alternative mechanism that provides partial ubiquitin pool misregulation. This result enabled some understanding of how proteins are degraded without ubiquitin [50]. Vanillin suppresses lung CSCs through Akt- and ubiquitin-dependent proteasomal degradation [16]. In contrast to vanillin, PCA may promote Akt degradation through a ubiquitin-independent proteasomal degradation pathway involving the Akt/Sox2 pathway (Figure 8).

This study demonstrates that PCA exhibits a strong CSC inhibitory effect via suppressing Akt/Sox2 signaling, but it has a limitation. It has a concentration limitation due to the use of high concentrations of PCA. PCA combined therapy is needed with existing chemotherapy or targeted therapy, which may improve the therapeutic effect and overcome the drug resistance. Finally, much research and development is required to confirm the possibility that PCA can be introduced into clinical practice as a new anti-CSC agent.

Here, we demonstrated that an active compound PCA, derived from *Artemisia princeps* through LAB fermentation, significantly inhibited breast CSC formation via Akt ubiquitin-independent degradation and downregulation of the transcription factor Sox2. Our findings support the anti-CSC effect of Akt/Sox2-targeting PCA, suggesting a novel utilization of PCA in BC therapy.

## 4. Materials and Methods

### 4.1. Cell Cultivation and Reagents

Human BC cell lines (MCF-7, MDA-MB-231, and HCC1937) were obtained from KCLB (Seoul, Republic of Korea), and the 4T1 (CRL-2539) mouse breast cancer cell line was acquired from ATCC (Manassas, VA, USA). MDA-MB-231 cells were cultured in Dulbecco’s modified Eagle’s medium (DMEM) supplemented with 1% penicillin/streptomycin and 10% fetal bovine serum (FBS) (Thermo Fisher Scientific, Waltham, MA, USA). MCF-7, HCC1937, and 4T1 cells were grown in RPMI medium (Thermo Fisher Scientific, Waltham, MA, USA). All media were supplemented with 10% FBS and 1% penicillin/streptomycin (Thermo Fisher Scientific, Waltham, MA, USA). Ultra-low attachment cluster tissue culture plates (6-well and 24-well) were purchased from Corning (New York, NY, USA). Protocatechualdehyde (PCA), CHX, and MG-132 were acquired from Sigma-Aldrich (St. Louis, MO, USA) and solubilized with DMSO.

### 4.2. Mammosphere Formation Assay

Cells (20,000 cells per well) were cultured in a cell floater plate (Corning, New York, NY, USA). All mammospheres were grown in MammoCult™ medium (STEMCELL Technologies, Vancouver, BC, Canada), supplemented with hydrocortisone and heparin on a cell floater plate. Mammospheres were cultured for 1 week. Mammosphere formation was quantified using the NICE program after image scanning [51].

### 4.3. Cell Proliferation Assay

Cell proliferation was performed using the EZ-cytox kit (Dogenbio, Seoul, Republic of Korea). MCF-7, MDA-MB-231, HCC1937, and 4T1 cells (2 × 10^3^/well) were plated into 96-well plates and administered with PCA dissolved in dimethyl sulfoxide (DMSO) for 24 h. After adding EZ-Cytox reagent to the 96-well plate, the cells were placed for 3–4 h at 37 °C. A_450_ nm was measured using a plate reader (VERSAmax microplate reader, Molecular Devices, Sunnyvale, CA, USA).

### 4.4. Colony Formation Assay

MDA-MB-231 (1 × 10^3^/well) was seeded on 6-well plates and cultured for 1 week. Following PCA treatment, cells were rinsed with 1× PBS and fixed with 4% paraformaldehyde for 10 min at RT and then stained with 0.03% crystal violet for 1 h. The colony formations were quantified using the Epson scanner (Tokyo, Japan) and the NICE software program (Version 1.1.0) [51].

### 4.5. Scratch Migration Assay

MDA-MB-231 cells were cultured in a 6-well plate and allowed to reach 100% confluency. A wound was made with a scratcher (SPL Life Sciences, Pocheon-si, Republic of Korea) on the cell layer, which was then treated with PCA. The scratch wound area was imaged at 16 h post-scratching using a microscope.

### 4.6. Cancer Stem Cell Isolation and Detection

The CD44^high^/CD24^low^ cell subpopulation was characterized as a marker of breast cancer stem cells. MDA-MB-231 cells (1 × 10^6^ cells/well) were harvested and incubated for 30 min with a buffer containing anti-human CD44 and CD24 antibodies (BD Biosciences, Franklin Lakes, NJ, USA). After incubation, fluorescence analysis of the cells was performed using flow cytometry (Accuri C6, BD Biosciences, Franklin Lakes, NJ, USA).

### 4.7. Apoptosis Assay

To measure apoptosis in mammospheres, the Annexin V Apoptosis Detection kit with PI (BD Biosciences, Franklin Lakes, NJ, USA) was utilized. Mammospheres treated with PCA (1 mM) were collected and dissociated using ACCUTASE (STEMCELL Technologies, Vancouver, BC, Canada). The dissociated cells were then cultured for 15 min at RT with a FACS buffer that contained Annexin V-FITC and PI. The cells were identified by a flow cytometer (Accuri C6, BD Biosciences, Franklin Lakes, NJ, USA).

### 4.8. ALDEFLUOR Assay

MDA-MB-231 cells were cultured in a 6-well plate for 24 h and treated with PCA. ALDH detection was performed using an ALDEFLUOR kit (STEMCELL Technologies, Vancouver, BC, Canada). These cells were trypsinized and stained following the vendor’s recommendations. Stained cells were analyzed using a flow cytometer. To determine specificity, a sample from each group was administered with 50 mmol/L DEAB, a specific ALDH inhibitor. Flow cytometry, using Accuri C6 (BD Biosciences, Franklin Lakes, NJ, USA) and NovoCyte 3000 (Agilent, Santa Clara, CA, USA), was used to analyze ALDH-positive cells. For cancer stem cell (CSC) analysis, single-cell isolations were performed using mouse tumor tissues, as described in the Section 4.11.

### 4.9. Western Blotting and Immunoprecipitation

Total proteins of BC and CSCs were extracted using RIPA lysis buffer containing a protease inhibitor and phosphatase inhibitor cocktail (GenDEPOT, Baker, TX, USA). Total proteins were resolved on sodium dodecyl sulfate–polyacrylamide gels and transferred onto polyvinylidene fluoride membranes (Millipore, Burlington, MA, USA). The membranes were blocked with 5% BSA for 1 h at RT and then incubated at 4 °C overnight with primary antibodies and horseradish peroxidase (HRP)-conjugated secondary antibodies (Santa Cruz Biotechnology, Dallas, TX, USA). Protein bands were visualized using the chemiluminescence method (GE Healthcare, Amersham, UK). We used a protein G immunoprecipitation kit to examine the interaction of Akt and ubiquitin (Thermo Fisher Scientific, Waltham, MA, USA). The beads were washed with washing buffer, and the samples were subsequently isolated by adding 2× SDS loading dye. The isolated samples were then subjected to Western blot analysis. The following antibodies were used: anti-pAkt, anti-Akt, anti-ubiquitin, anti-Sox2 (Cell Signaling Technology; CST, Danvers, MA, USA), and anti-β-actin (Santa Cruz Biotechnology, Dallas, TX, USA).

### 4.10. Mice

Adult female BALB/c mice (4–5 weeks old, ORIENT BIO, Seongnam-si, Republic of Korea) were used for animal experiments. All procedures were approved by the Institutional Animal Care and Use Committee (IACUC 24-026) of Ewha Womans University.

### 4.11. In Vivo Experiment

For subcutaneous tumor growth experiments, 1 × 10^6^ cells suspended in 100 μL 1× PBS were mixed with Matrigel (Corning, New York, NY, USA) and were inoculated into the hind flank of each mouse (*n* = 7). After 4T1 cell inoculation, mice with tumors received PCA injections (10 mg/kg, 1× PBS) every 3 days. Tumor growth was tracked by recording tumor sizes every 2–3 days, and tumor volumes were determined using the following formula: (length × width^2^)/2. Mice were sacrificed when the tumors reached approximately 4000 m^3^ in volume. Tumors were then excised, and a single-cell suspension was prepared by incubating small tumor pieces with ACCUMAX (STEMCELL Technologies, Vancouver, BC, Canada) for 30 min at room temperature. The tissue was strained through a 70 µm cell strainer to collect a single-cell suspension, which was subsequently analyzed by flow cytometry.

### 4.12. Statistical Analyses

Statistical analyses were performed using Student’s *t*-test to compare the differences between the two sample groups. For comparisons of multiple groups, one-way ANOVA was used, with Dunnett’s post-hoc test. All analyses were conducted using Prism 8.0 software (GraphPad Software Inc., San Diego, CA, USA) and presented as the mean ± standard deviation (SD), with statistical significance set at *p* < 0.05.

## 5. Conclusions

PCA derived from *Artemisia princeps* through LAB fermentation significantly suppresses mammosphere formation and decreases the CD44^high^/CD24^low^ subpopulation, ALDH1A-expressing cells, and Sox2 protein levels associated with self-renewal in breast CSCs by downregulating Akt and pAkt. This compound suppresses breast CSC formation via Akt ubiquitin-independent degradation and downregulation of the transcription factor Sox2. Our findings highlight the anti-CSC effect of Akt/Sox2-targeting PCA, suggesting an innovative application in BC therapy.

## Data Availability

The data presented in this study are available on request from the corresponding author.

## Supplementary Materials

The following supporting information can be downloaded at https://www.mdpi.com/article/10.3390/ijms26051811/s1.

## Abbreviations

BC	Breast cancer
CSC	Cancer stem cell
PCA	Protocatechualdehyde
TNBC	Triple negative breast cancer
