# Supplementary material for: Protocatechualdehyde Induced Breast Cancer Stem Cell Death via the Akt/Sox2 Signaling Pathway
Source: Int J Mol Sci. 2025 Feb 20;26(5):1811. doi: 10.3390/ijms26051811 (PMC11899452; doi:10.3390/ijms26051811)
Supplement: Supplementary file 1 [file ijms-26-01811-s001.zip › ijms-3456698-supplementary.pdf]

Supplementary Materials and Methods

Reagents. Silica gel 60A (35-75 micron) (ANALTECH, Newark, DE) resin was used for column chromatography. Thin layer chromatography (TLC) was conducted on a Kieselgel 60 F_{254} plate (MERK, Darmstadt, Germany). C18 silica gel resin was used for ODS column chromatography and was purchased from Sigma-Aldrich, Co. (St. Louis, MO). TLC plates were developed with solvent (chloroform and methanol (10:1 and 20:1)) and detected using a Spectroline UV lamp (Westbury, NY).

Plant materials. *Artemisia princeps* cultivated on Jeju Island, Republic of Korea were obtained from urban farmers (Seogwipo, Jeju, Republic of Korea). The leaves were rinsed with tap water, dried, and ground with a grinder (Hanil, Seoul, Korea). The voucher specimen (No. 2022_012) is deposited in the Department of Physiology, Inflammation-Cancer Microenvironment Research Center of Ewha Womans University, Seoul, Republic of Korea.

Lactic acid bacteria culture and fermentation using *Artemisia princeps* powder. *Lactobacillus rhamnosus* (ATCC 10863), an obligatory anaerobic homofermentative lactic acid bacteria, was purchased from the American Type Culture Collection (Manassas, Virginia, USA). Stock cultures of 1 mL were stocked at −80°C in Lactobacillus MRS medium (Difco, Detroit, USA) with 25% (v/v) glycerol. Flask experiments were performed in 1 L Erlenmeyer flasks containing 200 mL of the sterilized MRS medium cultured at 30°C and 37°C at 150 rpm for 24 h. MRS culture media containing 2% Artemisia powder was adjusted to pH 6.5. The pre-cultured L. rhamnosus broth was inoculated to sterilized MRS media containing 2% Artemisia and incubated at 37°C at 150 rpm for 0 or 5 days **(Figure S1)**.

Extraction and isolation. Dried artemisia powder (1kg) was incubated with MRS media (50L) containing L. rhamnosus, and the cultured media was centrifuged. Then, 50 L of centrifuged broth was extracted with 50 L of ethyl acetate. The ethyl acetate fraction was concentrated and solubilized with 100% methanol. Methanol extracts were mixed with distilled water and methanol parts were evaporated and the water part was recovered. Water extracts were purified using C-18 (ODS) column chromatography. Water-eluted parts showed inhibitory activity on mammosphere formation **(Figure S2)**. The concentrated MeOH fraction was applied to a silica gel column (25×350mm) and eluted with chloroform-methanol (10:1)**.** Each eluate was monitored via TLC; eight fractions were obtained and examined using mammosphere formation assays. The #6 fraction indicated inhibitory effects on mammosphere formation **(Figure S3)**. Therefore, this fraction was subjected to preparatory TLC twice (glass plate; 20×20cm) and developed in Hexanes:EA:Acetic acid (20:5:1) and chloroform-methanol (10:1) using a TLC glass chamber **(Figure S4 and S5)**. After development, plates were dried and detected by fluorescence under UV radiation (UV_{254nm} and UV_{365nm}). Individual bands were separated by scraping off the silica gel from the glass plate using a surgery knife and collected in a 15 mL conical tube. Then,

5 mL of methanol was added to the isolated gel, and the mixture was centrifuged and dried under a vacuum. Each fraction was obtained and examined using mammosphere formation assays. The active fraction was subjected to preparatory HPLC. HPLC analysis was examined using Shimadzu HPLC 20A (Shimadzu, Tokyo, Japan). Chromatographic separation was conducted using a shim-pack GIS-PREP-ODS 10×250mm C18 column. The sample was prepared and sieved through a 0.2μm syringe filter for HPLC analysis. The injection volume for HPLC isolation was 500 μL, the flow was 3 mL/min and the column temperature was room temperature at 220 nm. The mobile phase was composed of water (solvent A) and methanol (solvent B). For gradient elution, solvent B was initially set at 20%, increased to 60% at 20 min, and increased to 100% at 40 min. The purified sample was detected at a retention time of 33 minutes **(Figure S6)**. We concluded that the red box fraction contained a CSC inhibitor **(Figure S6)**. The bioassay-guided isolation procedure is summarized in Figure S7. The fraction was isolated using ethyl acetate extract, ODS column, silica gel, preparatory TLC, and HPLC. The purified sample was confirmed using HPLC and TLC **(Figure S6 and S7)**.

Structure analysis of the purified sample. The electrospray ionization (ESI) mass spectrum was measured on a QTRAP-3200 mass spectrometer (Applied Biosystems, Foster City, CA). Nuclear magnetic resonance (NMR) spectra were obtained on a JEOL JNM-ECA600, 600 MHz FT-NMR Spectrometer at 600 MHz for 1H NMR and at 150 MHz for ^{13}C NMR in CD_3OD. Chemical shifts are given in ppm (δ), with tetramethylsilane as the internal standard. For NMR spectra, two-dimensional NMR, such as ^{1}H-^{1}H COSY, HMQC, and HMBC, as well as one-dimensional NMR, such as ^{1}H NMR and ^{13}C NMR, was employed **(Figure S8, S9, S10, S11, and S12)**.

Supplementary Figures

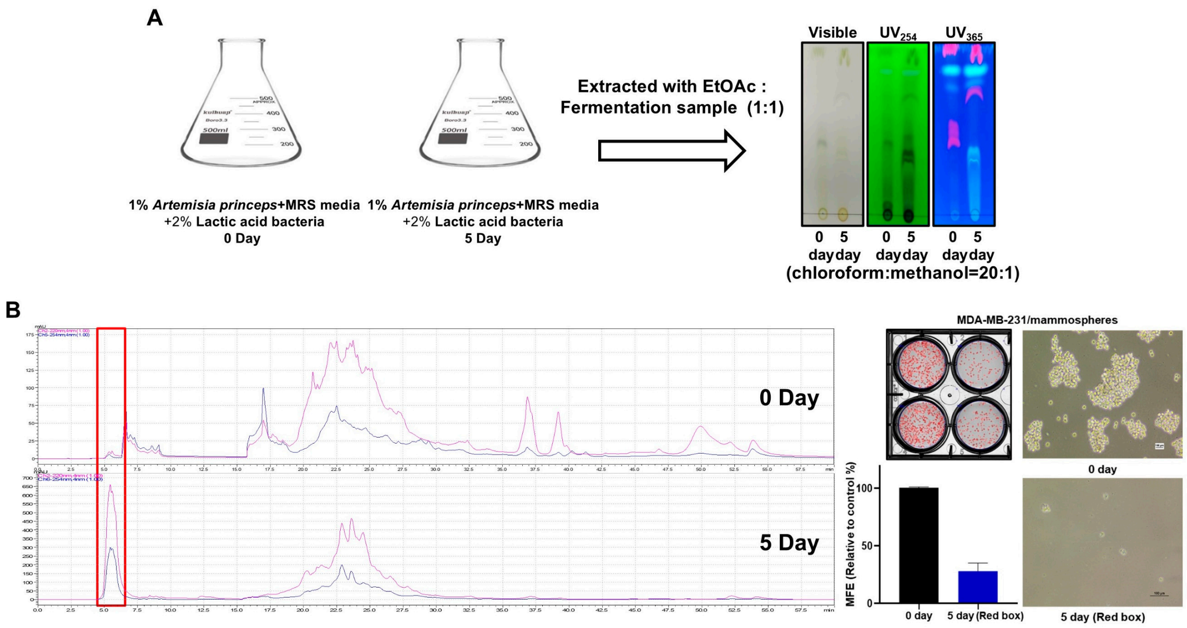

Figure S1. HPLC data of lactic acid bacteria cultured broth with *Artemisia princeps* extracts. (A) Lactic acid bacteria fermentation using *Artemisia princeps* extracts for 5 days. (B) HPLC data of lactic acid bacteria fermentation with *Artemisia princeps* extracts (0 and 5 day cultures).

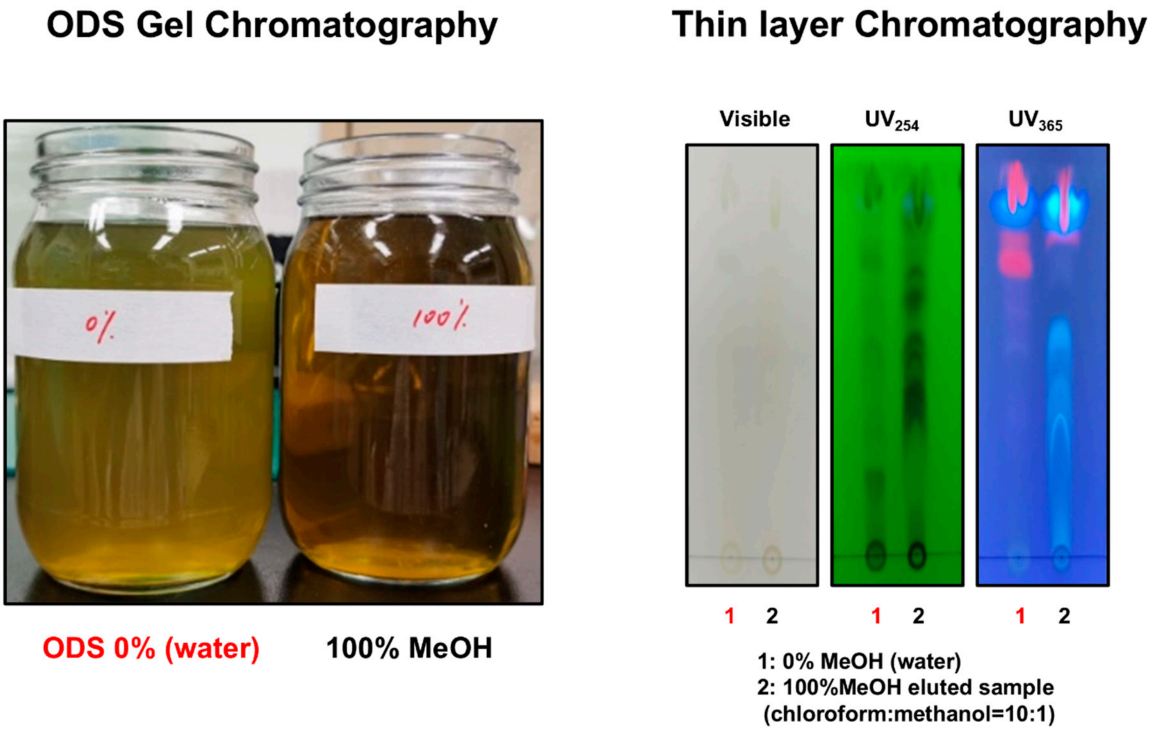

Figure S2. The isolation procedure of a CSC inhibitor derived from *Artemisia princeps*-fermented broth using ODS gel chromatography with water and MeOH. ODS open column chromatography is eluted with 0% (water) and 100% of MeOH using a sample. Thin layer chromatography analysis of the sample from ODS gel chromatography and the ODS samples were spotted and developed in chloroform: methanol (10:1). UV was used to detect the samples. Active fraction; water part.

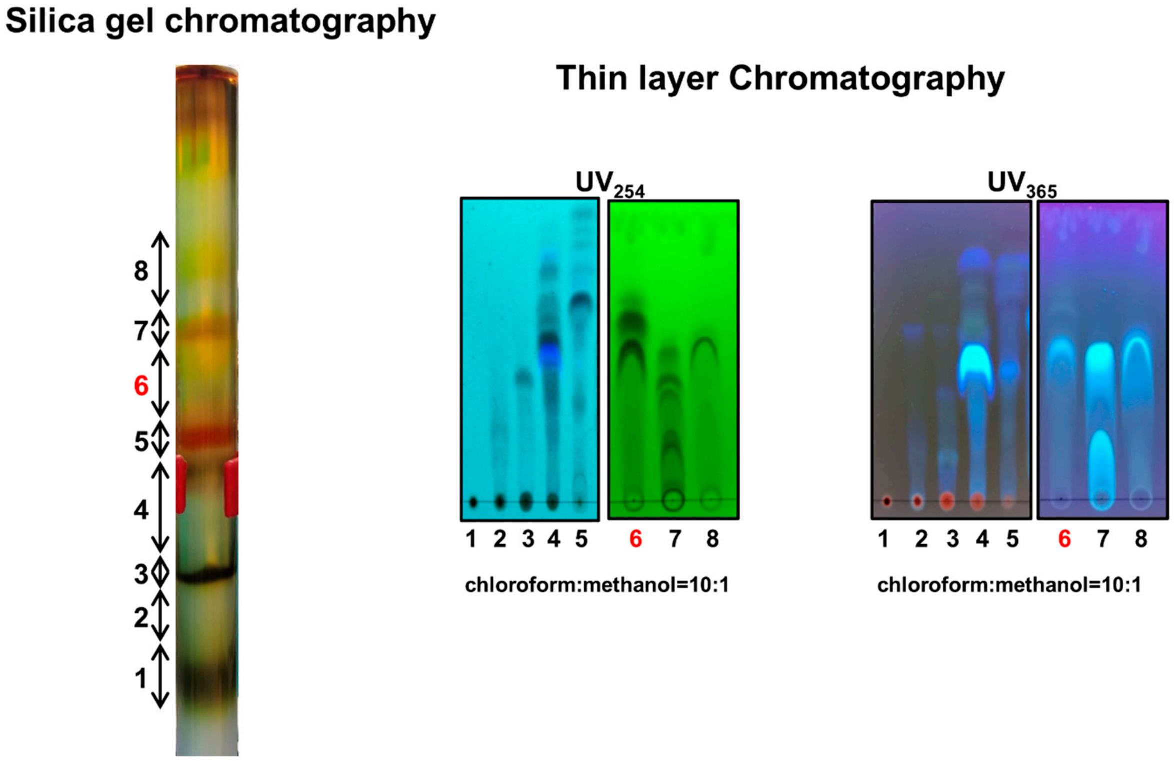

Figure S3. The isolation procedure of a CSC inhibitor with SiO_2 gel chromatography. The sample was purified by using SiO_2 gel chromatography with a solvent mixture [chloroform:methanol (10:1)]. Thin layer chromatography of the sample and the samples were spotted and developed with a solvent mixture (chloroform:methanol (10:1)). UV was used to detect the samples. Active fraction; #6.

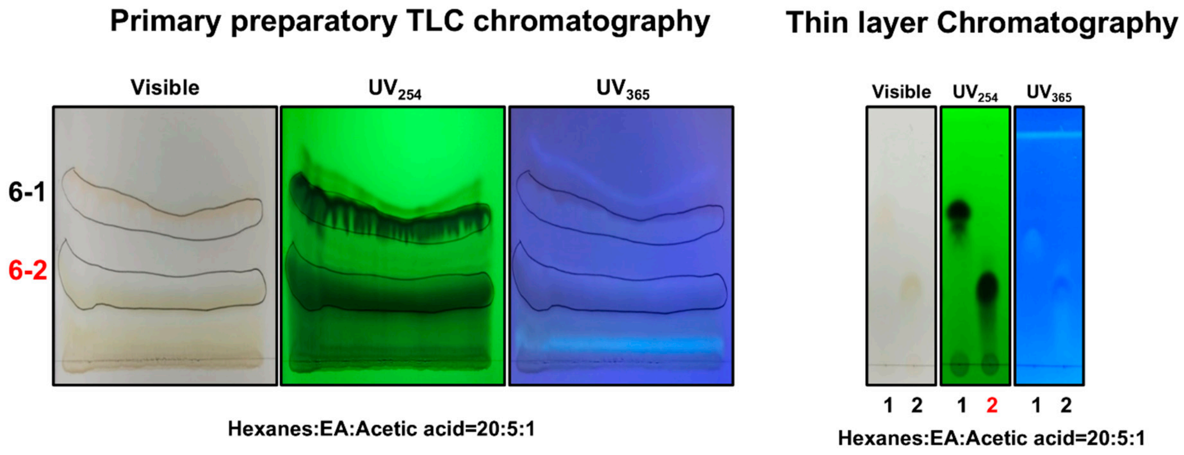

Figure S4. Isolation procedure of a CSC inhibitor from SiO_2 gel chromatography with preparative TLC with Hexanes:EA:Acetic acid (20:5:1). Active fraction; #6-2.

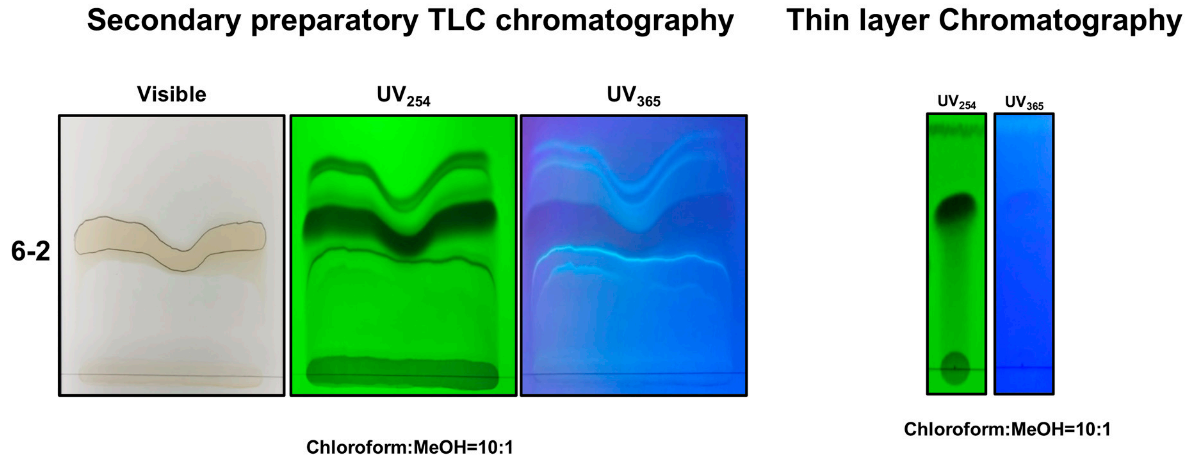

Figure S5. Isolation procedure of a cancer stem cell inhibitor with 2nd preparative TLC with $CHCl_3$: MeOH=10:1. Active fraction; #6-2.

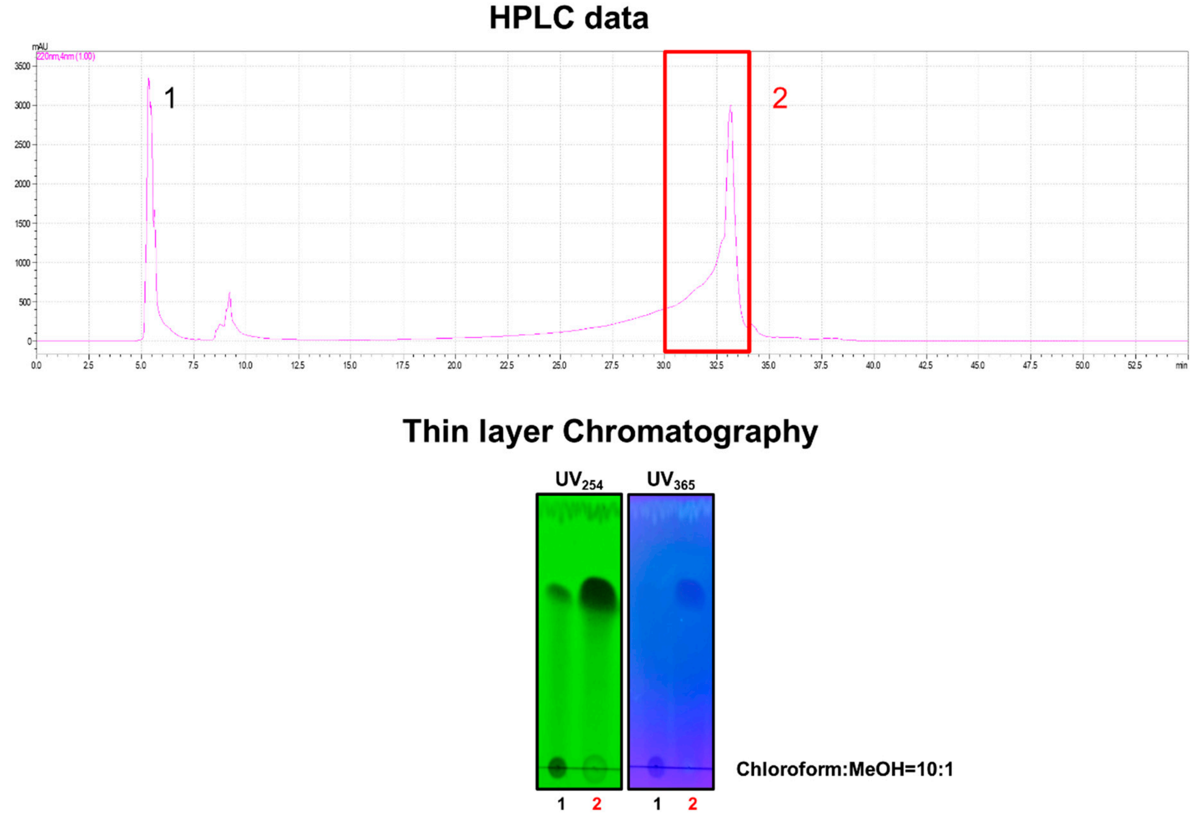

Figure S6. Major fractions using HPLC at two wavelengths. Samples were harvested based on 220 nm wavelength. HPLC chromatogram using purified sample. Active fraction; red box. TLC analysis of the isolated sample.

- ▼ **Fermentation (2% grinded plant and 4% lactic acid bacteria) for 5days**
- ▼ **Extraction (EtOAc : Fermentation sample = 1:1)**
- ▼ **ODS column chromatography eluted with water**
- ▼ **SiO_2 column chromatography eluted with $CHCl_3$: MeOH (10:1)**
- ▼ **Primary Prep. Thin layer chromatography (Hexanes: EA: Acetic acid=20:5:1)**
- ▼ **Secondary Prep. Thin layer chromatography ($CHCl_3$:MeOH=10:1)**
- ▼ **High performance liquid chromatography**

Figure S7. Isolation procedure of mammosphere formation inhibitor.

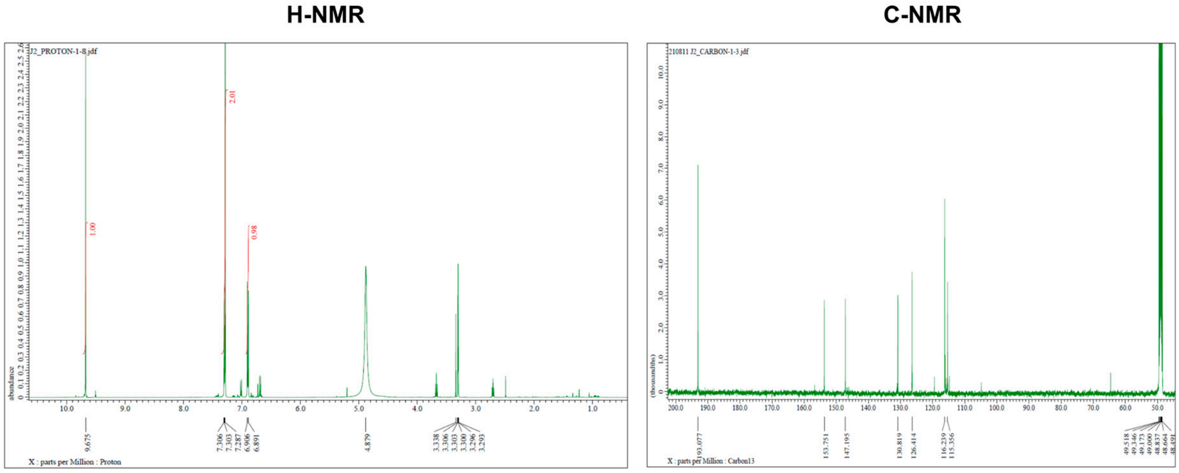

Figure S8. ^{1}H NMR spectrum in CD_3OD and ^{13}C NMR spectra of the isolated sample.

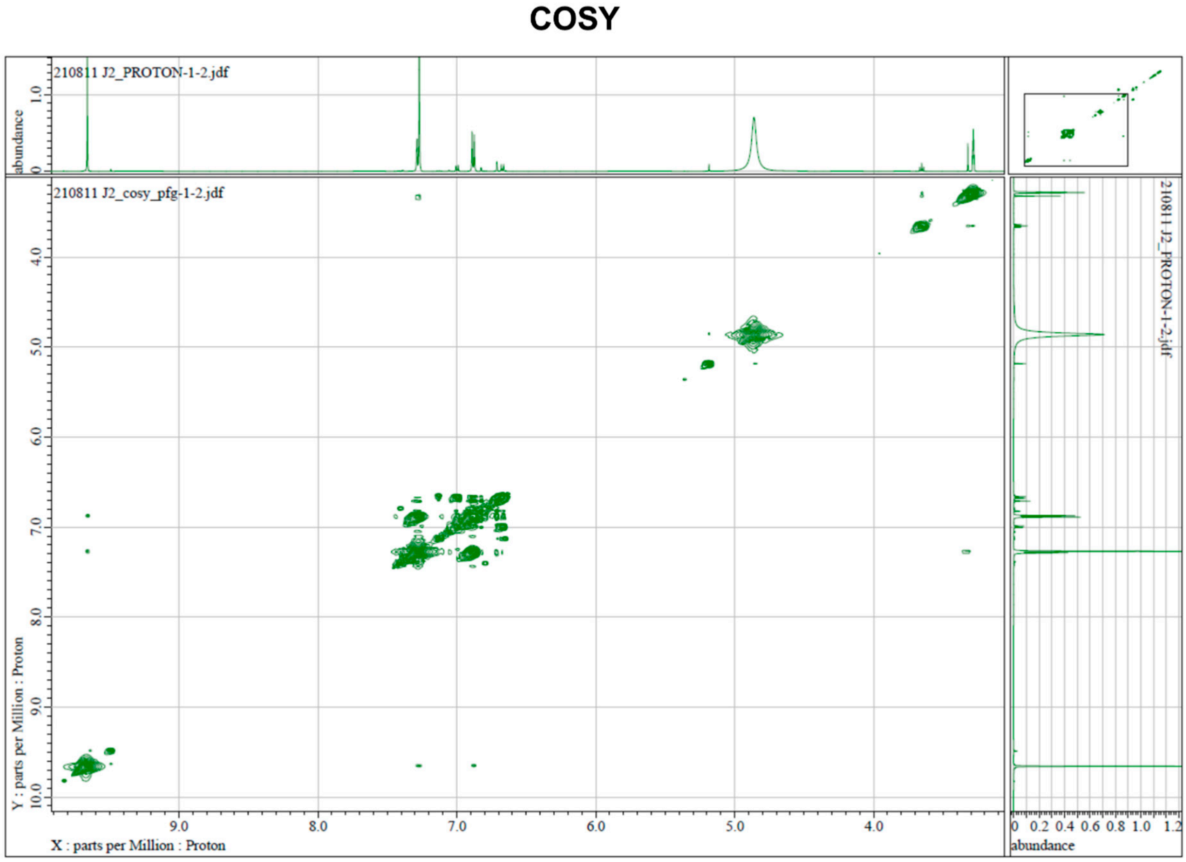

Figure S9. ^{1}H-^{1}H COSY spectrum of the isolated sample.

HMBC

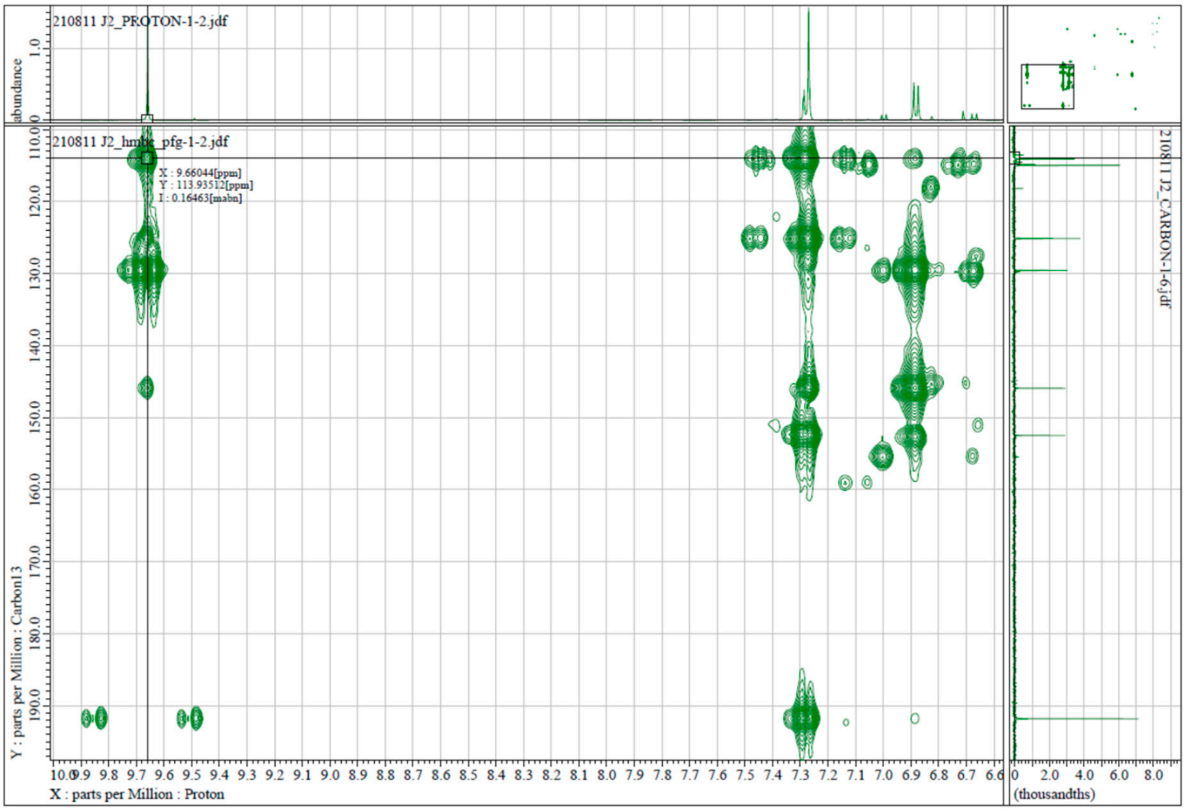

Figure S10. HMBC spectrum of the isolated sample.

HMQC

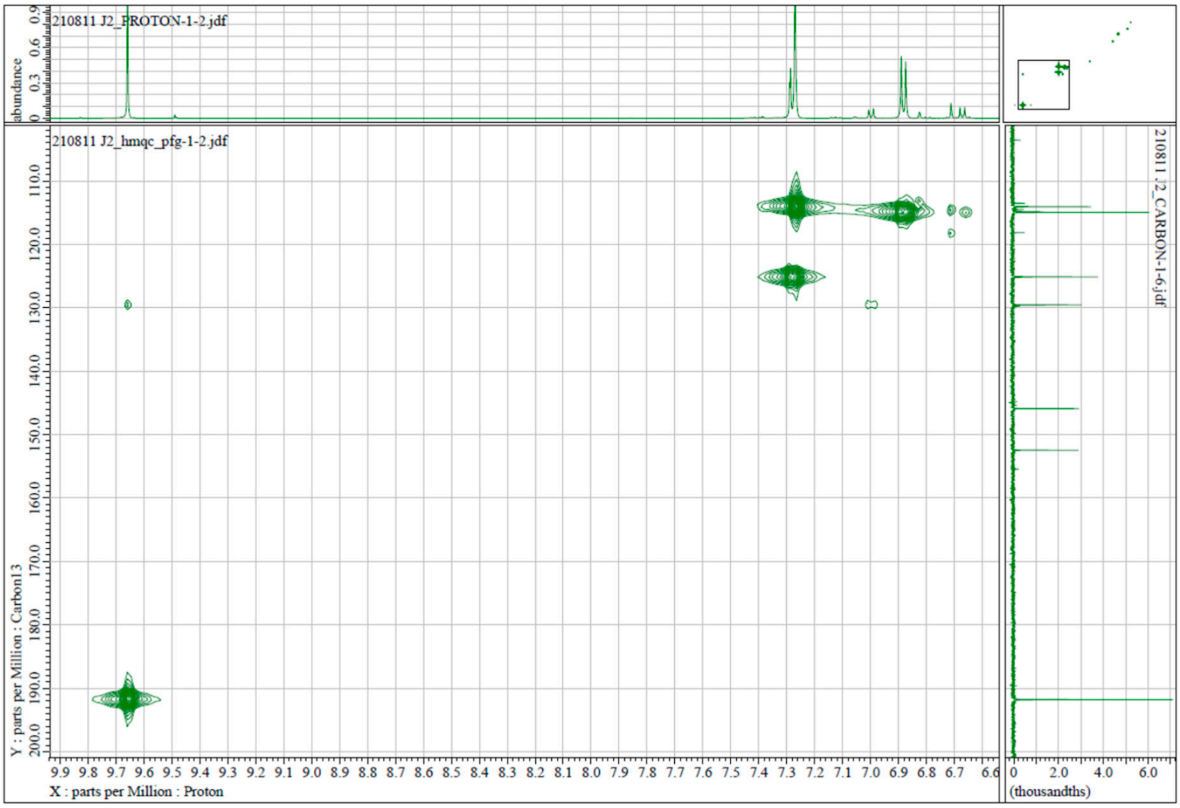

Figure S11. HMQC spectrum of isolated sample.

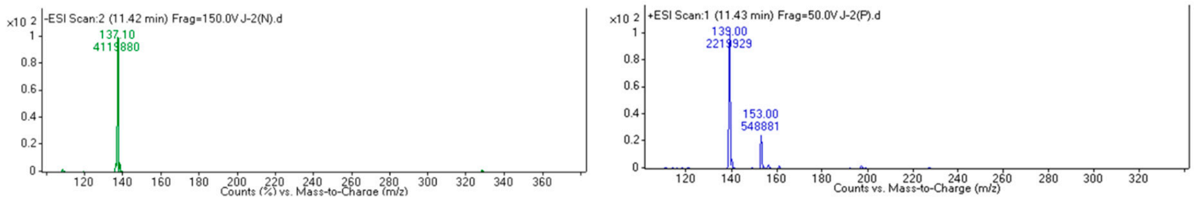

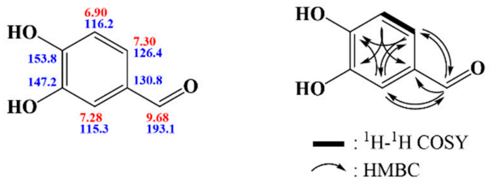

3,4-Dihydroxybenzaldehyde

Molecular formulation: $C_7H_6O_3$

Molecular weight: 138

Figure S12. ESI mass analysis and two-dimensional NMR correlations and ^{1}H and ^{13}C NMR peak assignments of the purified sample. The molecular mass was established as 138 Da by ESI spectrometry, which indicated quasi-molecular ion peaks at *m*/*z* 139 $[M+H]^+$.

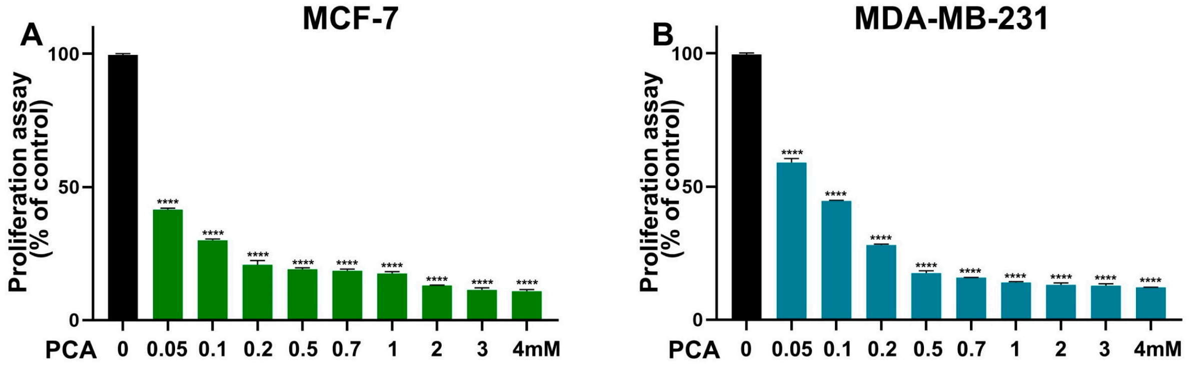

Figure S13. Effect of PCA on the proliferation of MCF-7 and MDA-MB-231 cells. (A, B) Anti-proliferative effects of PCA on MCF-7 and MDA-MB-231 cells, assessed using WST assays after treatment with increasing PCA concentrations for 3 days. Data are representative of three independent experiments. ****p < 0.0001 vs. control.

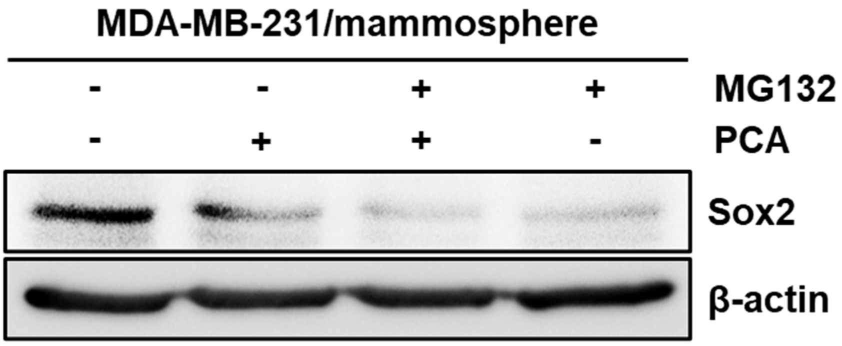

Figure S14. Mammospheres were incubated with MG132 and PCA (1mM) for 24 h and lysed for Western blot analysis for Sox2 protein. The treatment of cells with the proteasome inhibitor MG132 did not protect Sox2 from PCA-induced Sox2 degradation, suggesting that PCA did not enhance the proteasomal degradation of Sox2.
